# Narrow-band imaging and high-definition white-light endoscopy in patients with serrated lesions not fulfilling criteria for serrated polyposis syndrome: a randomized controlled trial with tandem colonoscopy

**DOI:** 10.1186/s12876-020-01257-4

**Published:** 2020-04-16

**Authors:** Fausto Riu Pons, Montserrat Andreu, Dolores Naranjo, Marco Antonio Álvarez-González, Agustín Seoane, Josep Maria Dedeu, Luis Barranco, Xavier Bessa

**Affiliations:** 1 Gastroenterology Department, Hospital del Mar, Passeig Marítim 25-29, 08003 Barcelona, Spain; 2 IMIM (Hospital del Mar Medical Research Institute), Barcelona, Spain; 3 Department of Medicine, Autonomous University of Barcelona, Barcelona, Spain; 4 Pompeu Fabra University, Barcelona, Spain; 5 Pathology Department, Hospital del Mar-IMIM, Barcelona, Spain

**Keywords:** Colonoscopy, Colonic polyps, Optical imaging, Narrow-band imaging

## Abstract

**Background:**

It is unknown whether narrow-band imaging (NBI) could be more effective than high-definition white-light endoscopy (HD-WLE) in detecting serrated lesions in patients with prior serrated lesions > 5 mm not completely fulfilling serrated polyposis syndrome (SPS) criteria.

**Methods:**

We conducted a randomized, cross-over trial in consecutive patients with prior detection of at least one serrated polyp ≥10 mm or ≥ 3 serrated polyps larger than 5 mm, both proximal to the sigmoid colon. Five experienced endoscopists performed same-day tandem colonoscopies, with the order being randomized 1:1 to NBI—HD-WLE or HD-WLE—NBI. All tandem colonoscopies were performed by the same endoscopist.

**Results:**

We included 41 patients. Baseline characteristics were similar in the two cohorts: NBI—HD-WLE (*n* = 21) and HD-WLE—NBI (*n* = 20). No differences were observed in the serrated lesion detection rate of NBI versus HD-WLE: 47.4% versus 51.9% (OR 0.84, 95% CI: 0.37–1.91) for the first and second withdrawal, respectively. Equally, no differences were found in the polyp miss rate of NBI versus HD-WLE: 21.3% versus 26.1% (OR 0.77, 95% CI: 0.43–1.38). Follow-up colonoscopy in nine patients (22%) allowed them to be reclassified as having SPS.

**Conclusions:**

In patients with previous serrated lesions, the serrated lesion detection rate was similar with NBI and HD-WLE. A shorter surveillance colonoscopy interval increases the detection of missed serrated polyps and could change the diagnosis of SPS in approximately one in every five patients.

**Trial registration:**

ClinicalTrials.gov NCT02406547, registered on April 2, 2015.

## Background

Colorectal cancer (CRC) is the second leading cause of cancer death in western countries [1, 2]. Conventional polyps are considered to be precursor lesions of sporadic CRC. Serrated polyps cause 20 to 35% of all CRC cases and are associated with interval CRC, especially when they involve microsatellite instability and the CpG island methylator phenotype [3]. These polyps are difficult to identify on endoscopy due to their location in the right colon, their sessile or flat morphology, and their pale color with mucus capping [4].

According to the World Health Organization (WHO) of 2010, serrated polyposis syndrome (SPS) was defined by the presence of one out of the following criteria: [5] 1) at least five serrated polyps proximal to the sigmoid colon, with two or more of these larger than 10 mm in diameter; 2) any number of serrated polyps occurring proximal to the sigmoid colon in an individual with a first-degree relative with SPS; and 3) more than 20 serrated polyps of any size distributed throughout the colon. In June 2019, a new update on the WHO SPS criteria was made with small differences between criteria 1 and 3, and the abandonment of criterion 2: 1) five or more serrated polyps proximal to the rectum, all being ≥5 mm in size, with at least two being ≥10 mm in size; and 2) more than 20 serrated polyps of any size distributed throughout the large bowel, with ≥5 being proximal to the rectum [6]. Therefore, patients with SPS are considered to be at increased risk of CRC. Because of the substantial risk of polyp recurrence in these patients, surveillance is mandatory with 1- to 2-yearly colonoscopies [7].

The European Society of Gastrointestinal Endoscopy has published the first Guideline of Advanced Endoscopic Imaging for the detection and differentiation of colorectal neoplasia and recommends conventional chromoendoscopy or narrow-band imaging (NBI) in patients with SPS (strong recommendation, low quality evidence) [8]. A pilot study in patients with SPS showed a significantly lower polyp miss rate with NBI than with high-definition white-light endoscopy (HD-WLE). However, this finding was not confirmed in a subsequent multicentre randomized trial by the same group [9, 10]. A randomized controlled trial in patients undergoing their first colonoscopy reported a non-significant trend toward detection of more proximal colon serrated lesions with NBI than with HD-WLE [11]. Finally, in a retrospective selected case series [12] of individuals in a population-based screening program with proximal serrated lesions at the baseline colonoscopy, colonoscopy reassessment within 1 year using chromoendoscopy and high-definition endoscopes led to a three-fold increase in the diagnosis of SPS.

We hypothesized that NBI could improve the detection of serrated polyps compared with HD-WLE in patients with prior relevant (larger than 5 mm) serrated lesions not fulfilling the SPS criteria. Therefore, this study was designed to compare the serrated polyp detection rate of NBI versus HD-WLE in a randomized, cross-over trial of tandem colonoscopy.

## Methods

### Study design and population

We performed a randomized, cross-over trial of tandem colonoscopy to compare NBI versus HD-WLE in the detection of serrated lesions in patients with previously resected serrated lesions not fulfilling SPS criteria between March 2015 and April 2016 at Hospital del Mar, Barcelona (Spain).

In compliance with the ethical guidelines of the Declaration of Helsinki, the study protocol was approved by the institutional review board of Parc de Salut Mar on 6 February 2015 (2014/5964/I) and was registered at ClinicalTrials.gov (NCT02406547). Written informed consent was obtained from all patients before study inclusion.

We included consecutive patients from the electronic database of the Endoscopy Unit who had undergone a complete baseline colonoscopy in the previous year, with any of the following findings: ≥ 1 serrated polyp proximal to the sigmoid colon larger than ≥10 mm in diameter; or ≥ 3 serrated polyps proximal to the sigmoid colon larger than 5 mm. All detected polyps were resected at the baseline colonoscopy. We used the proposed United Kingdom terminology, based on World Health Organization criteria, to define serrated lesions, including hyperplastic polyp (HP), sessile serrated lesions (SSL) with or without dysplasia (also known as sessile serrated polyp/adenoma), traditional serrated adenoma, and mixed polyp [13].

Patients were excluded if: 1) CRC was found at the baseline colonoscopy; 2) they did not attend follow-up or refused to provide informed consent; 3) they had a high risk of complications due to sedation (patients with high comorbidities considered by the American Society of Anesthesiologists to be grade IV and higher); 4) they had an incomplete colonoscopy; and 5) they had inadequate bowel preparation for colonoscopy defined by the Boston Bowel Preparation Score (BBPS): 0–1 points in any of the three segments of the colon [14, 15].

### Procedure and randomization

**Fig. 1:**
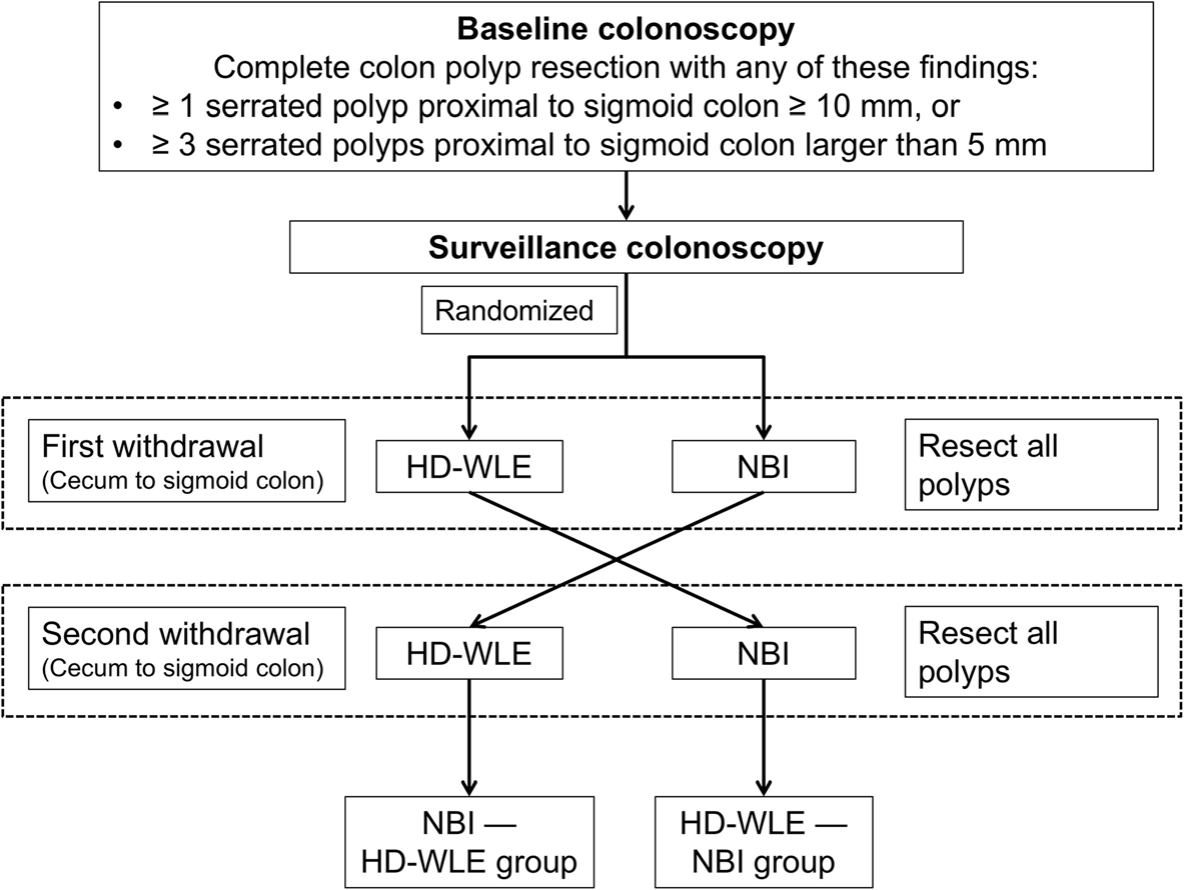
Study protocol. Back-to-back colonoscopy study with two randomized groups HD-WLE—NBI and NBI—HD-WLE

Patients were evaluated by the same endoscopist with tandem (“back-to-back”) colonoscopy. In this procedure, two withdrawals from the cecum to sigmoid colon were performed using the two techniques consecutively. At the first cecal intubation with HD-WLE, a sealed envelope was opened and each patient was randomly assigned 1:1 to the NBI—HD-WLE or the HD-WLE—NBI withdrawal group. All detected polyps were classified macroscopically and were resected at each withdrawal (Fig. 1). After detecting a polyp on NBI withdrawal, the endoscopist could switch from NBI to HD-WLE to perform the polypectomy and then resume NBI. Each polyp was recorded on a data sheet by nursing staff from the endoscopy room. All diminutive HPs resected from the rectosigmoid colon were excluded from the analysis. The endoscopists and study participants were blinded to the randomization list, which was computer-generated by the biomedical research consulting service of Hospital del Mar Medical Research Institute (IMIM), Barcelona.

Patients underwent a bowel preparation of 4 L split-dose polyethylene glycol solution. The level of sedation was monitored mostly by propofol alone or combined with midazolam and fentanyl at the discretion of the endoscopist. An anesthesia specialist was required in one patient.

High-definition colonoscopes (EVIS EXERA III CV-190; Olympus Inc., Tokyo, Japan) were used by five experienced senior endoscopists (experience with > 4000 colonoscopies). These endoscopes utilize high-definition images to produce NBI with a brighter quality than that obtained with 180 series colonoscopies [16].

Histological specimens were collected and revised by blinded expert gastrointestinal pathologists. They were not informed which sample was received from the study protocol.

Patient data included age, sex, weight and height, comorbidities, personal and family history of CRC and smoking habits. Data from the study protocol included median time to evaluation, time for each withdrawal (excluding time of therapeutic polypectomy), lesion histology, localization, morphology, Paris classification [17], and the BBPS. Localization of polyps was divided into five anatomical segments: cecum, ascending, transverse (including the hepatic and splenic flexures) and descending colon (from the splenic flexure to the sigmoid colon).

### Outcome measures

The primary outcome was to measure the number of serrated polyps detected with NBI and HD-WLE. Secondary outcomes were the number of missed lesions from the first technique used for each group (NBI or HD-WLE), defined as the proportion of polyps detected on the second withdrawal relative to the number of polyps found during the two examinations, and to assess the number of patients reclassified as having SPS after the scheduled colonoscopy.

### Statistical analysis and sample size

All data were prospectively recorded and stored in a database. Continuous variables are expressed as the mean and standard deviation (SD) or median and interquartile range (IQR) for skewed data and categorical variables as frequencies (%). Continuous variables were compared using the t-test if normally distributed and the Mann-Whitney U test if not. Categorical variables were compared using the chi-square test or Fisher exact test. Associations with the polyp, serrated polyp and adenoma miss rates were calculated using odds ratios with 95% confidence intervals. Two-tailed *P* values < 0.05 were considered to be statistically significant. Statistical analyses were performed using STATA/IC software (version 13.1. StataCorp Lp, College Station, TX, United States).

In a previous study in SPS patients, NBI showed an increase of up to three-fold in the detection of HPs compared with HD-WLE. Assuming a polyp miss rate on colonoscopy of around 22% [18]. we hypothesized that NBI would decrease this ratio from 22 to 7% [9]. To detect this difference with an 80% power and a significance level of 0.05, 81 polyps were required. We assumed that at least two polyps would be found in these patients and therefore 41 patients (81/2) were required for the study.

## Results

### Baseline characteristics

**Fig. 2:**
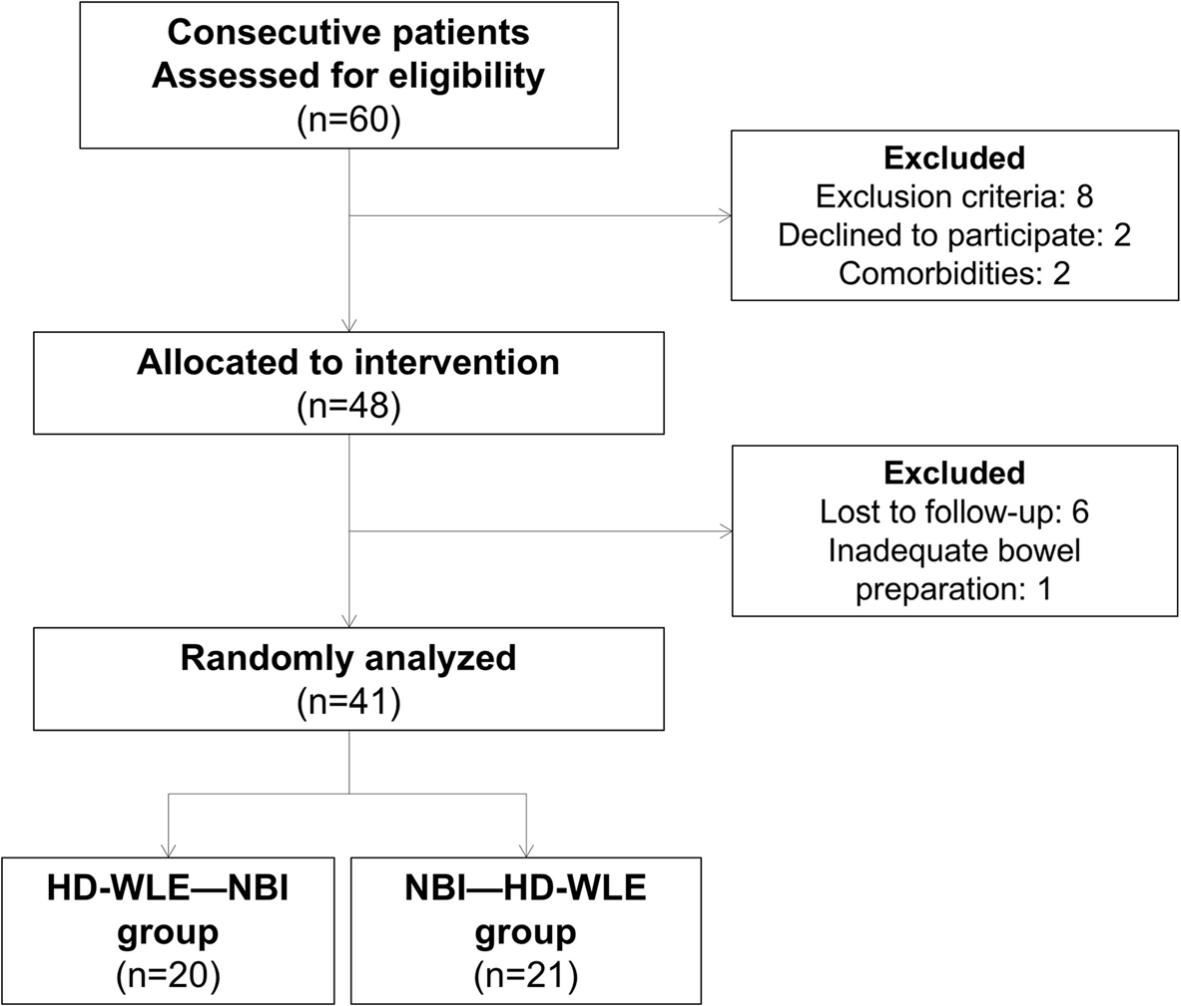
CONSORT flow chart diagram of study selection

Of 60 patients assessed for eligibility, 12 patients were excluded before intervention (eight due to exclusion criteria, two declined to participate, and two patients had high comorbidity) and seven patients were excluded after allocation (six were lost to follow-up and one patient had inadequate bowel preparation). Forty-one patients were finally included and randomly assigned: 20 patients in the HD-WLE—NBI group and 21 in the NBI—HD-WLE group (Fig. 2).

The patients’ baseline characteristics are shown in Table 1. Patients were mostly men (53.7%) with no major comorbidities. Smoking habits were reported by 25/41 patients (60.9%), and current smokers (34.2%) had a smoking history of a median of 17.5 pack-years. A family history of CRC was present in 12 (29.3%) patients with no personal history of CRC or family history of SPS. The indication for the first colonoscopy was mainly due to CRC screening based on a positive fecal immunochemical test in 19 patients (46.3%), followed by polyp surveillance in 10 patients (24.4%).

**Table 1:** Patient baseline characteristics

Total number of patients (n)	41
Mean age, years (SD)	59.6 (8.6)
Male sex (%)	53.7
BMI (Kg/m^2^, SD)	26.9 (4.2)
Diabetes mellitus (n, %)	4 (9.8)
Smoking habits (n, %)	
Non-smokers	16 (39.0)
Former smoker	11 (26.8)
Current smoker	14 (34.2)
Smoking pack-years (median, IQR)	17.5 (14–31)
Comorbidities (n, %)	
None	21 (51.2)
Obesity	2 (4.9)
Cardiovascular	9 (22.0)
Respiratory	4 (9.8)
Any cancer	2 (4.9)
Multiple pathology	3 (7.3)
Family history of CRC (n, %)	12 (29.3)
Personal history of CRC (n, %)	0
Personal history of abdominal surgery (n, %)	17 (41.5)
Colonoscopy indication (n, %)	
CRC screening	19 (46.3)
Surveillance for colonic neoplasia	10 (24.4)
Diagnosis or clinical symptoms	6 (14.6)
Family history of CRC screening	5 (12.2)
Therapeutic colonoscopy	1 (2.4)
Baseline inclusion criteria (n, %) ^a^	
One or more serrated polyp larger than 10 mm	26 (63.4)
Three or more serrated polyps	15 (36.6)
Type of colonoscope at baseline examination	
165 Olympus series	4 (9.8)
180 Olympus series	14 (34.1)
190 Olympus series	23 (56.1)
Mean time from baseline to colonoscopy review (months, 95% CI)	6.7 (5.2–8.3)

*BMI* body mass index, *SD* standard deviation, *IQR* interquartile range, *CRC* colorectal cancer, *CI* confidence interval

^a^All polyps were proximal to the sigmoid colon

At the baseline colonoscopy, 26 of the 41 patients (63.4%) had at least one serrated polyp larger than 10 mm and 15 of the 41 patients (36.6%) had three or more serrated polyps proximal to the sigmoid colon. In the latter 15 patients, both inclusion criteria were present in 10 patients.

### Comparison of NBI and HD-WLE

Patient characteristics were similar in the two groups (Table 2). There were no major complications due to the colonoscopy intervention. The mean time from baseline to colonoscopy review was 6.7 months (95% CI, 5.2–8.3) with no differences between groups. The median time required for the first withdrawal was similar (11.1 vs 12.9 min, for HD-WLE and NBI, respectively, *P* = 0.2). Statistically significant differences were found for the second withdrawal, which was 2 min longer for NBI in the HD-WLE—NBI group than in HD-WLE in the NBI—HD-WLE group, (9.1 vs 7.1 min, *P* = 0.05).

**Table 2:** Characteristics between groups

	HD-WLE—NBI (n = 20)	NBI—HD-WLE (n = 21)	*P* value
Males (n, %)	9 (45)	13 (61.9)	0.35
Age (years, mean)	59.9	59.3	0.84
BMI (Kg/m^2^, mean)	26.1	27.7	0.24
Median number of detected polyps on 1st withdrawal (n, IQR)	2.5 (1–4)	3 (2–8)	0.15
Median size of resected polyps on 1st withdrawal (mm, IQR)	3 (2–4)	3 (2–4)	0.8
Mean time 1st withdrawal (min, SD)	11.1 (3.9)	12.9 (4.4)	0.2
Mean time 2nd withdrawal (min, SD)	9.1 (3.7)	7.1 (2.1)	0.05
Mean time from baseline colonoscopy (months, 95% CI)	7.2 (4.4–10.0)	6.2 (4.5–7.9)	0.53
Colonoscope at baseline examination (n, %)			0.99
165 Olympus series	2 (10.0)	2 (9.5)	
180 Olympus series	7 (35.0)	7 (33.3)	
190 Olympus series	11 (55.0)	12 (57.1)	

*HD-WLE* high-definition white light endoscopy, *NBI* narrow-band imaging, *BMI* body mass index, *SD* standard deviation, *IQR* interquartile range

**Fig. 3:**
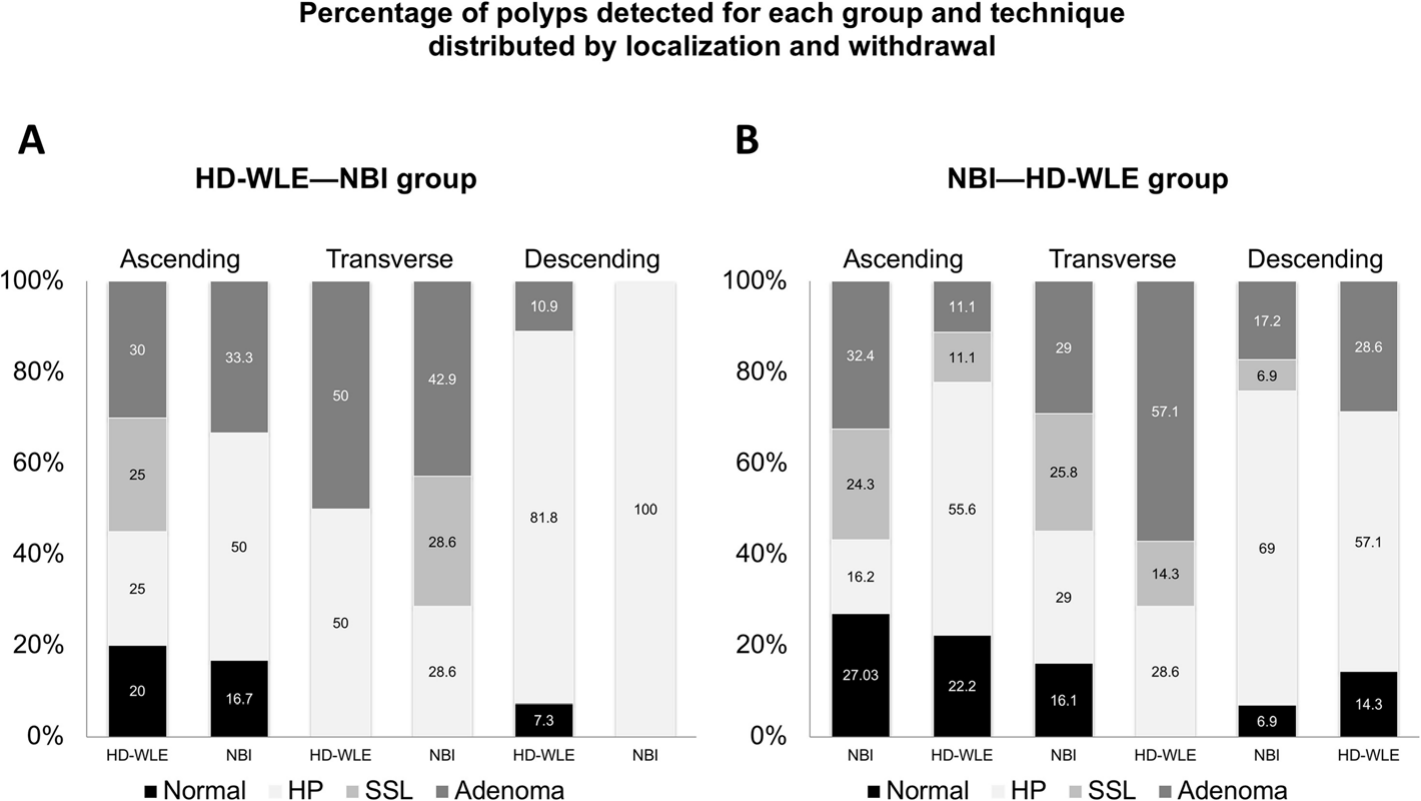
Percentage of polyps detected for each group (HD-WLE—NBI and NBI—HD-WLE) and technique distributed by localization and withdrawal. The x-axis shows withdrawals with the two techniques for each anatomical location. The first column shows the first withdrawal with HD-WLE for HD-WLE—NBI group (**a**) or with NBI for NBI—HD-WLE group (**b**) and the second column shows the results of the second withdrawal with the opposite. The y-axis shows the polyp detection rate distributed by histology. Abbreviations: HD-WLE: high-definition white light endoscopy; NBI: narrow-band imaging; SSL: sessile serrated lesion; HP: hyperplastic polyp

A total of 246 suspected polyps were detected with a median size of 3 mm (IQR: 2–4). Histology was obtained in 228: 113 (49.6%) HPs, 28 (12.3%) SSL with no dysplasia, 57 (25.0%) adenomas, 29 (12.7%) with normal histology and one lipoma (0.4%) (Table 3, Fig. 3). The median number of polyps detected was similar with HD-WLE and NBI on the first withdrawal for each group, HD-WLE—NBI group: 2.5 polyps (IQR: 1–4) versus NBI—HD-WLE group: 3 polyps (IQR: 2–8), *P* = 0.15. No differences were observed in the detection rate of serrated lesions by NBI versus HD-WLE: 47.4% vs 51.9% (OR 0.84, 95% CI: 0.37–1.91) for the first and second withdrawal, respectively.

**Table 3:** Histology of all resected polyps in the two groups

	HD-WLE—NBI group			NBI—HD-WLE group			
	HD-WLE (n, %) First withdrawal	NBI (n, %) Second withdrawal		NBI (n, %) First withdrawal	HD-WLE (n, %) Second withdrawal	Total (n, %)	
Hyperplastic	55 (64.7)	12 (52.2)		35 (36.1)	11 (47.8)	113 (49.6)	
Sessile serrated lesion	5 (5.9)	2 (8.7)		19 (19.6)	2 (8.7)	28 (12.3)	
Adenoma	17 (20.0)	7 (30.4)		26 (26.8)	7 (30.4)	57 (25)	
Normal	8 (9.4)	2 (8.7)		17 (17.5)	2 (9.3)	29 (12.7)	
Other (lipoma)	–	–		–	1 (4.4)	1 (0.4)	
Total (n)	85	23	***P*** **= 0.23**	97	23	228	***P*** **= 0.32**

*HD-WLE* high-definition white light endoscopy, *NBI* narrow-band imaging

Thirty-four relevant serrated lesions, defined as any polyp larger than 5 mm, were detected proximal to the sigmoid junction, with no differences between groups (*P* = 0.33). Seven serrated lesions were larger than 10 mm: four in the HD-WLE—NBI group and three in the NBI—HD-WLE group. Only two were detected with HD-WLE on the first withdrawal from the HD-WLE—NBI group, and the remaining five were detected by NBI (three on first withdrawal and two on the second).

No differences were observed in the polyp miss rate between HD-WLE and NBI, calculated as the percentage of missed polyps at the second withdrawal with regard to the first: 21.3 and 26.1% (OR 0.77, 95% CI: 0.43–1.38) for NBI and HD-WLE, respectively. The serrated polyp miss rate (including proximal hyperplastic and serrated lesions, both larger than 5 mm) was 25.0 and 28.6% (OR 0.83, 95% CI: 0.19–3.63) for NBI and HD-WLE, and the adenoma miss rate was 21.2 and 29.2% (OR 0.65, 95% CI: 0.2–2.13) for NBI and HD-WLE, respectively.

After reassessment, nine patients were reclassified as having SPS based on 2010’s criteria and the new 2019’s criteria: six patients had criterion 1 (at least five proximal serrated polyps, all being ≥5 mm in size, two of which were larger than 10 mm in diameter), and three patients had criterion 2 (more than 20 serrated polyps of any size distributed throughout the colon, with ≥5 being proximal to the rectum). These patients represented 21.95% (9/41) of the individuals reassessed (Table 4).

**Table 4:** Characteristics of patients with reassessment of SPS criteria

Patient	Baseline colonoscopy	Follow-up colonoscopy	SPS 2010’s criteria	SPS 2019’s criteria
**#1**	2 SSL (10 and 15 mm) 1 TVA HGD (35 mm) 1 TA LGD (< 10 mm)	1 SSL (10 mm) 4 HP (3, 3, 6 and 8 mm) 1 TA LGD (< 10 mm)	No. 1	No. 1
**#2**	2 SSL (11 and 12 mm) 1 SSL (8 mm) 1 HP (7 mm)	1 SSL (5 mm) 2 HP (4 and 9 mm) 1 TA LGD (< 10 mm)	No. 1	No. 1
**#3**	2 SSL (5 and 8 mm) 1 HP (6 mm) 3 TA LGD (< 10 mm)	2 SSL (10 and 15 mm) 4 HP (2, 4, 6 and 10 mm) 1 TA LGD (< 10 mm)	No. 1	No. 1
**#4**	2 SSL (13 and 15 mm) 2 SSL (8 and 10 mm) 1 TVA HGD (20 mm)	2 HP (6 and 10 mm) 2 TA LGD (< 10 mm)	No. 1	No. 1
**#5**	1 SSL (10 mm) 1 SSL (6 mm) 4 HP (4, 5, 5 and 8 mm) 3 TA LGD (< 10 mm)	1 SSL (20 mm) 5 SSL (2, 2, 4, 4 and 8 mm) 3 HP (3, 5 and 8 mm) 3 TA LGD (< 10 mm)	No. 1	No. 1
**#6**	1 SSL (< 10 mm) 11 HP (< 10 mm) 4 TA LGD (< 10 mm)	1 SSL (< 10 mm) 9 HP (< 10 mm) 4 TA LGD (< 10 mm)	No. 3	No. 2
**#7**	15 HP (< 10 mm) 1 TSA (15 mm) 5 TA LGD (< 10 mm)	1 SSL (< 10 mm) 7 HP (< 10 mm)	No. 3	No. 2
**#8**	1 SSL (< 10 mm) 1 TSA (11 mm) 10 HP (< 10 mm)	2 SSL (< 10 mm) 8 HP (< 10 mm)	No. 3	No. 2
**#9**	1 SSL (20 mm) 1 HP (4 mm) 2 TA LGD (< 10 mm)	1 SSL (12 mm) 2 SSL (5 and 6 mm) 3 HP (3, 3 and 5 mm) 1 TA LGD (< 10 mm)	No. 1	No. 1

*SPS* serrated polyposis syndrome, *SSL* sessile serrated lesion, *HP* hyperplastic polyp, *TVA* tubulovillous adenoma, *TA* tubular adenoma, *TSA* traditional serrated adenoma, *LGD* low grade dysplasia, *HGD* high grade dysplasia. The World Health Organization (WHO) for diagnosis SPS of 2010’s criteria: criterion no.1 refers to at least five serrated polyps proximal to the sigmoid colon, two of which are larger than 10 mm in diameter; SPS criterion no. 2 refers to any number of serrated polyps occurring proximal to the sigmoid colon in an individual with a first-degree relative with SPS; SPS criterion no. 3 refers to more than 20 serrated polyps of any size distributed throughout the colon. The WHO’s 2019 criteria: SPS criterion no. 1 refers to five or more serrated lesions/polyps proximal to the rectum, all being ≥5 mm in size, with at least two of which are larger than 10 mm in diameter; SPS criterion no. 2 refers to more than 20 serrated lesions/polyps of any size distributed throughout the colon, with ≥5 being proximal to the rectum

## Discussion

In this randomized cross-over study, we found that the use of NBI versus HD-WLE in patients with a history of relevant serrated lesions not fulfilling SPS criteria was not superior in detecting polyps, including serrated lesions. However, reassessment of these patients, independently of the evaluated technique, allowed one-fifth of them to be reclassified as having SPS. In addition, we found no differences in the polyp miss rate on first evaluation with NBI or HD-WLE (21.3% versus 26.1%).

This prospective study confirms previously published results on the non-superiority of NBI in the detection of serrated lesions compared with HD-WLE. A multicenter randomized cross-over trial of NBI and HD-WLE in 51 patients with SPS concluded that the two techniques were similar, showing a non-significant trend in the polyp miss rate: 20% with NBI (95% CI, 15–27) versus 29% with HD-WLE (95% CI, 22–36), *P* = 0.065. This difference was driven by the lower HP miss rate with NBI [10]. Moreover, a recent randomized controlled trial with NBI and HD-WLE using the Olympus 190 series for the detection of proximal colon serrated lesions in routine colonoscopy patients showed no statistically significant differences, but found a trend toward higher detection of serrated lesions with NBI than with HD-WLE (0.51 versus 0.39 serrated lesions per patient, *P* = 0.085) [11].

One of the most salient findings of our randomized controlled study is that after the scheduled follow-up colonoscopy, nine patients (22%) were reassessed as meeting SPS criteria, both the 2010’s and the recently updated 2019’s criteria, with important clinical implications in these patients. A similar finding was observed in a retrospective study of a fecal immunochemical test (FIT)-based CRC screening program [12], in which a 1-year reassessment colonoscopy of arbitrary selected participants with previous serrated polyps detected by chromoendoscopy and high-definition endoscopes substantially improved SPS detection in those with proximal serrated lesions.

Without endoscopic reevaluation, most societies, such as the US Multi-Society Task Force, the European Society of Gastrointestinal Endoscopy and recently the British Society of Gastroenterology, classify these patients as high risk and recommend they undergo surveillance colonoscopy at a 3-yearly interval (patients with at least one sessile serrated lesion or a traditional serrated adenoma ≥10 mm or with a dysplastic component (weak recommendation, low quality evidence) [7, 19, 20]. The results of our study emphasize the clinical implications of the addition of high-quality endoscopic reassessment in patients with relevant serrated lesions, allowing around 20% of them to be reclassified as having SPS and to undergo close surveillance.

Of note, our inclusion criteria (more than one serrated lesion ≥10 mm or more than three serrated lesions proximal to the sigmoid colon) were not based on pre-established criteria but involved a high-risk group according to clinical guidelines. In the aforementioned retrospective study [12], the factors associated with SPS reassessment were five or more proximal serrated lesions or two or more sessile serrated polyps  ≥  10  mm. When we applied these criteria to our study, only 12 of our 41 (29.3%) patients met the threshold for endoscopic reevaluation, and two of the nine patients with SPS were misclassified.

These results have implications for participants in CRC screening programs. In a study of five European cohorts, the rate of any serrated lesion was between 15.1 and 27.2% (1.1 and 2.6% with at least one serrated lesion larger than 10 mm), with SPS being diagnosed in 0.4 to 0.8% after a follow-up colonoscopy (1:127 individuals were diagnosed with SPS during colonoscopy follow-up in the Spanish FIT-based CRC screening cohort) [21]. Identification of patients with SPS has important implications related to the higher risk of CRC. Indeed, in a large multicenter study conducted in Spain, CRC was diagnosed in 15.8% of all patients with SPS with a cumulative risk of CRC of 1.9% at 5 years [22]. Therefore, identifying patients who require close surveillance is essential to decrease the incidence of CRC.

This study has some limitations. First, it is a single-center study in a small number of patients based on a sample size calculation. However, given that we found no statistically significant differences, it is likely that a larger study would probably yield similar results. In addition, the study was underpowered to show differences in the detection of large SSL > 10 mm. Secondly, back-to-back colonoscopies were performed by the same endoscopist. Although it would have been better if two different endoscopists had performed each withdrawal colonoscopy, we considered this study design to be the most effective way to reduce interobserver variation [18] and to be the most feasible in practice. The main advantages are that the presence of a single endoscopist in the room eliminates other confounders such as differences in skill or experience between distinct endoscopists. Therefore, the withdrawal time and polyp/adenoma detection rate were identical for each endoscopist. Finally, although NBI is designed for use at a close distance from the mucosa to visualize vascular and pit patterns, our study was not designed to evaluate the accuracy of the endoscopists in characterizing detected lesions.

## Conclusion

Our study shows that in patients with previously resected relevant serrated lesions there were no differences in the use of NBI or HD-WLE in the detection of serrated lesions, with the two techniques having a similar polyp miss rate. We suggest that NBI is an option in clinical practice but not necessarily as a matter of routine. Notably, the results of our study emphasize the importance of an exhaustive examination in patients with suspicion of relevant serrated lesions. Although further studies are needed to define the exact threshold for endoscopic reassessment of patients with relevant serrated lesions, the results of our prospective study, which confirm the substantial percentage of polyp miss rate and the clinical implications of SPS, suggest that a short reassessment colonoscopy interval would be suitable in these patients.

## Data Availability

The dataset generated and analyzed during the study is stored in a secure localized database but is available from the corresponding author in an anonymous format on reasonable request.

## Abbreviations

BBPS: Boston Bowel Preparation Score
BMI: Body mass index
CI: Confidence interval
CRC: Colorectal cancer
FIT: Fecal immunochemical test
HD-WLE: High-definition white-light endoscopy
HGD: High grade dysplasia
HP: Hyperplastic polyp
IMIM: Hospital del Mar Medical Research Institute
IQR: Interquartile range
LGD: Low grade dysplasia
NBI: Narrow-band imaging
SD: Standard deviation
SPS: Serrated polyposis syndrome
SSL: Sessile serrated lesions
TA: Tubular adenoma
TSA: Traditional serrated adenoma
TVA: Tubulovillous adenoma
